# Bidirectional Lipid Droplet Velocities Are Controlled by Differential Binding Strengths of HCV Core DII Protein

**DOI:** 10.1371/journal.pone.0078065

**Published:** 2013-11-01

**Authors:** Rodney K. Lyn, Graham Hope, Allison R. Sherratt, John McLauchlan, John Paul Pezacki

**Affiliations:** 1 National Research Council of Canada, Ottawa, Ontario, Canada; 2 Department of Chemistry, University of Ottawa, Ottawa, Ontario, Canada; 3 Medical Research Council-University of Glasgow Centre for Virus Research, Glasgow, Scotland, United Kingdom; University of North Carolina School of Medicine, United States of America

## Abstract

Host cell lipid droplets (LD) are essential in the hepatitis C virus (HCV) life cycle and are targeted by the viral capsid core protein. Core-coated LDs accumulate in the perinuclear region and facilitate viral particle assembly, but it is unclear how mobility of these LDs is directed by core. Herein we used two-photon fluorescence, differential interference contrast imaging, and coherent anti-Stokes Raman scattering microscopies, to reveal novel core-mediated changes to LD dynamics. Expression of core protein’s lipid binding domain II (DII-core) induced slower LD speeds, but did not affect directionality of movement on microtubules. Modulating the LD binding strength of DII-core further impacted LD mobility, revealing the temporal effects of LD-bound DII-core. These results for DII-core coated LDs support a model for core-mediated LD localization that involves core slowing down the rate of movement of LDs until localization at the perinuclear region is accomplished where LD movement ceases. The guided localization of LDs by HCV core protein not only is essential to the viral life cycle but also poses an interesting target for the development of antiviral strategies against HCV.

## Introduction

Once thought to be a benign storage organelle, the lipid droplet (LD) has gained attention for its involvement in many cell functions including cellular signaling, membrane organization, and trafficking [1], [2], [3]. LDs are primarily composed of triglycerides (TG) and cholesterol esters in a hydrophobic core that is surrounded by a phospholipid monolayer [4]. Additionally, the LD surface is coated in proteins that facilitate cellular signaling interactions, control the access of metabolic enzymes, and influence LD movement within the cell [4], [5], [6], [7].

Biochemical and live-cell imaging analyses have shown LD movement is microtubule-dependent [8], [9], [10], [11] and is facilitated by motor proteins that move along microtubules radiating from the microtubule organizing center (MTOC) [9]. LDs are shuttled towards the MTOC in a dynein-mediated retrograde (minus-end motion) manner, while movement away from the MTOC is mediated by kinesin motors in an anterograde manner (plus-end motion) [12], [13], [14], [15]. Immunofluorescence studies of peroxisomes in *Drosophila* have demonstrated that both motors are localized on cargo at the same time [16], with evidence of bidirectional LD movement shown in human hepatocytes [17]. Accordingly, bidirectional LD transport is likely coordinated to direct net movement to meet cellular demands [11], [18].

Productive hepatitis C virus (HCV) infection is tightly linked to hepatic lipid metabolism and requires direct interactions with LDs for propagation [19], [20]. HCV infection is the leading cause of liver disease, affecting 2 to 3% of the global population [21], [22]. More than half of HCV infections result in dense accumulation of LDs, a phenotype commonly known as liver steatosis [23], [24], [25]. There is strong support for a direct relationship between HCV and LDs, highlighting LDs as a key host organelle involved in pathogenesis [19], [25], [26], [27], [28], [29], [30], [31], [32].

**Figure 1:**
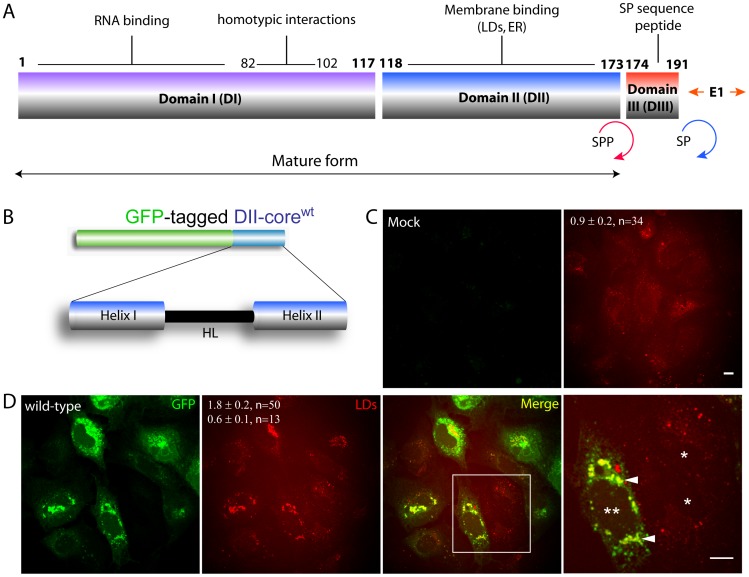
GFP-tagged DII-core^wt^ colocalizes with LDs. (A) Schematic representation of HCV core protein. Distinct interactions belong to each of the three core protein domains. The mature and immature forms are also shown, and are generated by the two host proteases: signal peptidase (SP, blue), and signal peptide peptidase (SPP, red). (B) GFP-tagged DII-core^wt^ contains the membrane binding domain consisting of two α-amphipathic helices separated by a hydrophobic loop. (C-D) CARS microscopy imaging of LDs in Huh-7 cells expressing GFP-tagged DII-core^wt^. All images were collected approximately 20 hours after Huh-7 cells were transfected with (D) DII-core^wt^ and (C) without DII-core^wt^, which contained only the lipofectamine transfection reagent. (C) Lipid volumes measured by voxel analysis for mock Huh-7 cells are shown in the CARS image. (D) CARS imaging captures DII-core^wt^ induced LD biogenesis and redistribution towards the perinuclear region. The two values in panel 2 represent the average LD volume for cells expressing DII-core^wt^ (top value, double asterisks) and non-expressing DII-core cells (single asterisks) within the same field of view (bottom value) as measured by voxel analysis. The error represents standard error of the mean. The n represents the amount of cells quantified for LD density. This experiment was conducted under two biological replicates. Panel 4 is a magnified image selected by a region of interest from the merged image to project a clearer view of colocalization between DII-core^wt^ and LDs. All scale bars represent 10 µm.

HCV is a single-stranded, positive-sense RNA virus encoding a polyprotein that is processed into 3 structural and 7 non-structural proteins (reviewed in [33]). Of particular interest is the core protein, which forms the viral capsid, since it also accumulates on the LD surface [17], [34], [35]. The mature form of core is generated through sequential cleavage by two host proteases (Figure 1A) [36], [37], [38], [39], [40]. This mature form of core, which consists of two domains, termed I and II, translocates from the ER to the LD surface, with domain II (DII) involved in LD binding [41], [42]. The core-LD interaction is essential in the HCV lifecycle, since its disruption eliminates viral particle assembly [39], [43], [44], [45]. Although the fate of lipids contained in the core-bound LDs is unclear, host lipids are used in virtually every step of the viral lifecycle and function as viral dependant post-translational modifications for both host and viral proteins [23], [26], [30], [46], [47], [48]. Ultimately, these interactions also permit the establishment of platforms involved in viral assembly through LD-associated membrane interactions [25], [49].

HCV-induced changes in LD morphology and dynamics can be investigated by coherent anti-Stokes Raman scattering (CARS) microscopy. CARS is a molecular imaging tool that uses high excitation laser pulses to enhance the vibrational resonances of chemical bonds, and can be specifically tuned to generate high-contrast images of select organelles and/or drug molecules in the cell [50], [51]. As such, the C-H bonds of long fatty acid chains that are densely packed in LDs generate excellent signal contrast for LD imaging [52], [53]. Furthermore, since CARS is a label-free technique, video-rate imaging of LD dynamics is possible without the use of chemical stains that may perturb the cellular environment.

We have previously shown changes in LD morphology as well as mobility in human hepatoma cells after 48 hours of expression of a HCV genotype 3a form of core (core3a) [17], [35]. In addition, core3a expression increased cellular LD volume before the LD migrated towards the perinuclear region. In this study, we focus on LD dynamics at an earlier time point of core protein expression using a GFP-tagged DII of JFH1 core protein (DII-core) to visualize core protein’s localization on the LD surface [45], in addition to LD particle tracking. This method enabled particle tracking of DII-core bound LDs, along with LDs in non-DII-core expressing cells, and allowed their rates of transport in the cell to be monitored. We also evaluated single amino acid mutations in the LD binding domain of core to determine whether LDs are dynamically modified by the binding strength of DII-core. Overall, our findings provide new insight into the effects of HCV core protein on LD dynamics. Uncovering details of the HCV life cycle not only expands our understanding of this important pathogen, but also offers alternative targets for the development of host-targeted therapeutics.

## Results

### Expression of GFP-tagged DII-core^wt^ Increases Cellular LD Volume

Changes in LD dynamics that are induced by bound fluorescently-tagged proteins can be monitored by simultaneous two-photon fluorescence (TPF) and CARS microscopy. With this method, the dynamics of both bound and unbound LD populations can be compared within the same cell and/or field of view. Previously, we showed that an N-terminal GFP-tagged construct of DII-core from the JFH1 strain (Figure 1B, DII-core^wt^) was capable of LD colocalization [45]. These results are supported herein by simultaneous TPF and CARS microscopy, which show DII-core^wt^ retained a colocalized pattern with cytoplasmic LDs in Huh-7 cells (Figure 1D).

It was also possible to make qualitative and quantitative comparisons between LD dynamics and mobility in DII-core^wt^ expressing and non-expressing cells under one single field of view. We observed a distinct change in LD density for cells expressing DII-core^wt^ (Figure 1D, single vs. double asterisks), while LDs observed in non-DII core expressing cells were comparable to mock Huh-7 cells (Figure 1C & D, single asterisks). Voxel analysis used to calculate LD density revealed a 3-fold increase in LD density of DII-core^wt^ expressing cells to non-expressing cells under the same field of view (Figure 1D). We also observed a change in LD localization in cells expressing DII-core^wt^, with LD clusters located at the perinuclear region (Figure 1D, arrowheads). Our images show that clusters of LDs were absent in the mock and non-DII-core expressing cells (Figure 1C & D, single asterisks). This suggests that DII-core^wt^ is capable of inducing LD migration towards the perinuclear region much like full-length core [45], likely by affecting interactions with motor proteins that are involved in LD motility [35]. Importantly, we observed that GFP did not disrupt DII-core binding to LDs, indicating that GFP-tagged DII-core is suitable to study the dynamics of LD mobility.

### DII-core^wt^ Modulates LD Dynamics when it is bound to the LD Surface

**Figure 2:**
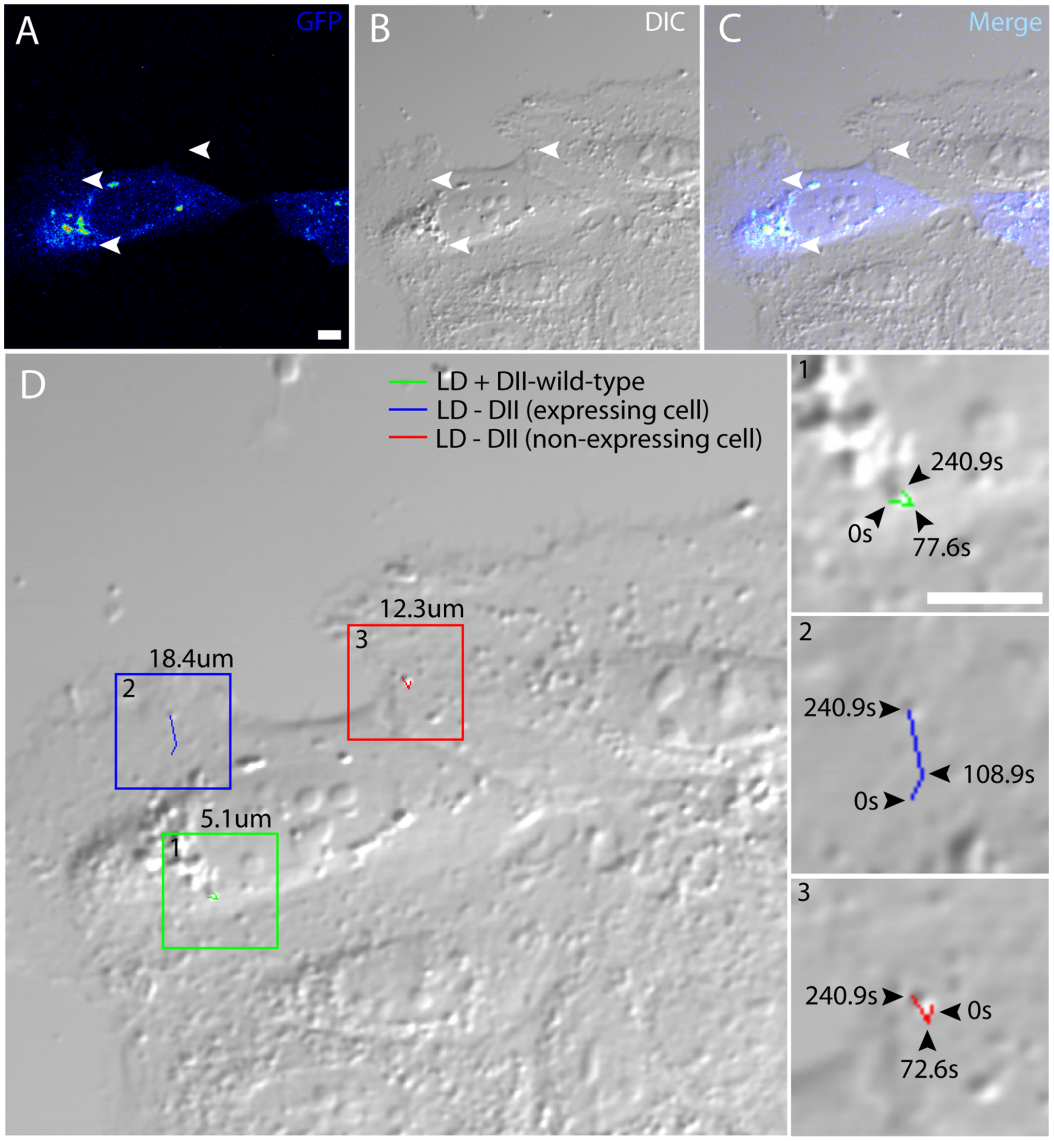
DII-core^wt^ coated LDs are particle tracked using simultaneous TPF and DIC microscopy. This is a representative image of DII-core^wt^ expressed in Huh-7 cells. Three individual LDs with dissimilar environments were selected (A–C, white arrows), and their trajectories were measured to calculate the overall distances traveled. (D) A larger DIC image of (B) includes boxes to identify each LD trajectory (inset 1–3). The value above each box (D) indicates their overall travel distances for (1) DII-core^wt^ coated LD, (2) non DII-core^wt^ coated LD within the same cell, (3) and a LD in an adjacent cell not expressing DII-core^wt^. Each LD trajectory is magnified to demonstrate the LD track with selective freeze frame time-intervals representing the LD position at their indicated times. Due to frequent bidirectional movements, the displayed trajectories represent a general movement path, and does not portray total distance. All of the LDs are tracked according to the same start and end time. All scale bars represent 10 µm.

Since DII-core^wt^ behaves similarly to naïve full-length core protein [45], we assessed whether the interaction between DII-core^wt^ and LDs affected LD mobility. DII-core^wt^ expressing cells contain populations of naïve and DII-core^wt^-bound LDs. By simultaneous TPF and differential interference contrast (DIC) imaging, the trajectories of LDs from both populations can be tracked by following LD movements with and without overlap of the DII-core^wt^ GFP tag. It is important to note that LD mobility may potentially be affected by factors including cell passage number, biological replicate and cell confluency. To circumvent this, in every experiment that was conducted, the LD measurements acquired from Huh-7 cells expressing DII-core^wt^ were directly compared with LD measurements from a mock sample of the same biological replicate. We have previously shown that LDs in full-length core expressing cells were motile, but travel at half the speeds by comparison to mock LDs [17]. With GFP-tagged DII-core^wt^ expressing cells, we observed a similar pattern, and showed that DII-core^wt^ coated LDs traveled with an average speed of approximately 40.3 nm/sec compared to LDs in mock-treated Huh-7 cells, which traveled at 67.2 nm/sec (Table 1). To compare these values, we divided the average speeds of DII-core^wt^ coated LDs by LDs in mock cells and observed a ratio of 0.60. To illustrate these changes more clearly, a representative image was captured from a time-course movie (Figure 2A–C, arrowheads) that tracked spatially unique LDs under different expression conditions within the same field of view. For example, the general trajectories of LD mobility for individual DII-core^wt^ coated and non-coated LDs in the same cell are illustrated (Figure 2D, box 1 vs box 2, inset 1 vs inset 2). As expected, non-coated LDs travelled a longer distance. Additionally, LDs in an adjacent non-expressing cell traveled further than LDs that are bound to DII-core^wt^ (Figure 2D, box 3 and inset 3). Furthermore, the presence of HCV non-structural proteins, which are recruited to LDs during the viral lifecycle and are required to form the membranous web [54], do not affect the ability of DII-core^wt^ to induce changes in LD speeds and travel distances (Figure S1).

**Table 1:** Mean speeds and travel distances of DII-core coated LDs compared to LDs in [mock transfected] Huh-7 cells^a^.

Construct	Distance traveled (µm)	Mean speed (nm/s)	Ratio
Wild-type (n = 39) [n = 138]	9.7±1 [16.2±0.6]	40.3±3 [67.2±2]	0.60
G161F (n = 51) [n = 53]	5.8±0.4 [12.4±0.7]	24.3±2 [51.7±3]	0.47
G161L (n = 19) [n = 60]	7.4±0.8 [12.8±0.8]	30.8±3 [53.3±3]	0.58
G161S (n = 39) [n = 46]	8.9±0.7 [11.5±0.6]	36.8±3 [47.7±3]	0.77
G161A (n = 51) [n = 50]	10.2±0.9 [13.1±0.8]	42.2±4 [54.5±3]	0.77

a The mean speeds and overall travel distances of LDs are compared in DII-core expressing Huh-7 cells and LDs in mock cells (enclosed in square brackets). The error represents standard error of the mean. The n represents the number of LDs from eight or more cells in different fields of view, assessed by particle tracking for both mutant (enclosed in round brackets) and mock samples (enclosed in square brackets). Live-cell imaging was conducted for duration of four minutes acquiring each frame at rate of 1.65 sec/frame. To minimize variability for LD speeds, all of the experiments that directly compared DII-core coated LDs to LDs in a mock sample were observed in cells of the same biological replicate. The ratios were calculated by dividing the mean speed of DII-core coated LDs by LDs in the mock cells.

### The Binding Strength of DII Dictates the Overall LD Mean Speeds and Travel Distances

**Figure 3:**
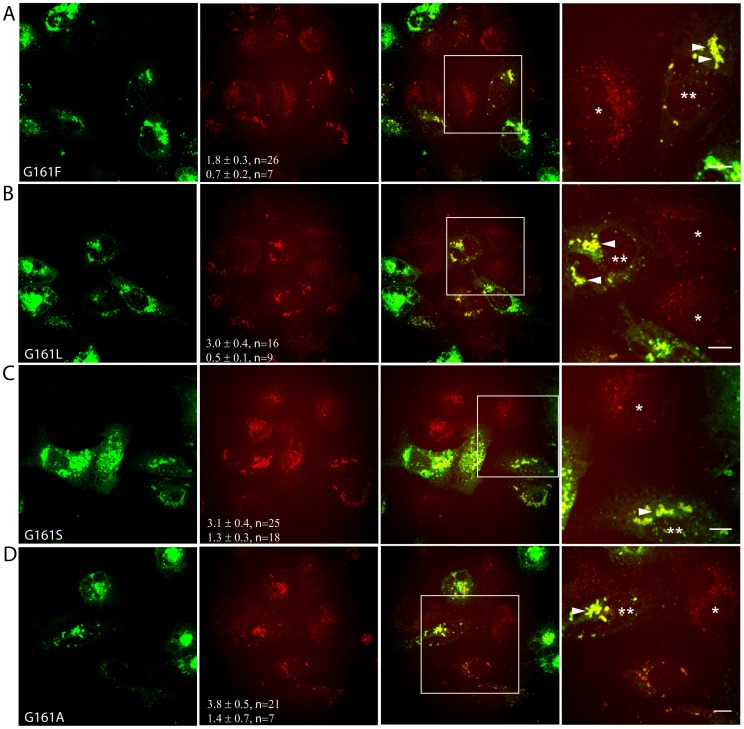
Simultaneous CARS and TPF microscopy captures LD changes induced by single amino acid mutations in GFP-tagged DII-core^161^ expressing Huh-7 cells. All images were collected approximately 20 hours after Huh-7 cells were transfected with (A) DII-core^G161F^, (B) DII-core^G161L^, (C) DII-core^G161S^, and (D) DII-core^G161A^. CARS imaging identifies DII-core^161^ induced LD biogenesis and redistribution towards the perinuclear region under the expression of all DII-core^161^ mutants (A–D, panel 4, arrowheads). The two values in panel 2 represent the average LD volume for cells expressing DII-core^161^ (top value, double asterisks) and non-expressing DII-core^161^ cells (single asterisks) within the same field of view (bottom value) as measured by voxel analysis. The error represents standard error of the mean. The n represents the number of cells that were quantified for LD density. This experiment was conducted under two biological replicates. Panel 4 is a magnified image selected by a region of interest from the merged image to project a clearer view of colocalization between DII-core^161^ mutants and LDs. The scale bar represents 10 µm.

Since DII-core^wt^ can modulate LD mobility, we postulated that single amino acid modifications targeting the interaction between DII-core and LD interface could variably affect LD dynamics. To evaluate this, we mutated glycine 161 (G161) of the DII second α-helix to alter hydrophobicity, since it is conserved among all six HCV genotypes and is predicted to lie within the cytosol-lipid interface [28], [55], [56]. We have found that increasing the hydrophobicity of residue 161 increases the binding strength, while a hydrophilic substitution decreased binding strength of DII-core to LDs (Filipe et al., manuscript in preparation). To ensure that the DII-core^161^ mutations did not disrupt targeting, we first evaluated whether these DII-core^161^ mutants colocalized with LDs in Huh-7 cells. As shown in Figure 3, GFP-tagged DII-core^161^ mutants colocalized with LDs, and induced LD migration to the perinuclear region, as is observed for DII-core^wt^. Expression of these mutants also increased LD volumes 3–5 fold.

To investigate the effect of LD binding strength of DII-core on LD dynamics, we next determined LD speeds of the G161 mutants. Generally, cells expressing DII-core^161^ mutants with large hydrophobic side chains had slower LD mean speeds than wt; however, all were consistently lower than the mean LD speeds of their mock samples (Table 1). DII-core^G161F^, for example, exhibited approximately half the mean LD speed of mock, with a ratio calculated to be 0.47. Conversely, mean LD speeds of DII-core^G161S^ and DII-core^G161A^ were faster than wt, with both ratios calculated to be 0.77. The LD mean speeds measured for DII-core^161^ mutant expressing cells gave a general trend that appeared to depend upon the binding strength of DII with LDs (Table 1).

Similar to what was observed for DII-core^wt^, populations of core-coated and naïve LDs also exist within DII-core^161^ mutant expressing Huh-7 cells. Therefore, we can directly measure LD trajectories of differential travel distances for individual LDs within the same cell, depending on whether the LD is bound to DII-core^161^. We used DII-core^G161F^ expressing Huh-7 cells as a representative image to evaluate both LD populations (Figure S2A–C). The trajectories of DII-core^G161F^ coated LDs ultimately traveled shorter distances compared to non DII-core^G161F^ coated LDs within the same cell (Figure S2D, box 1 vs box 2, inset 1 vs inset 2). Correspondingly, LDs in non DII-core^G161F^ expressing cells also resulted in longer travel distances (Figure S2D, box 3 and inset 3). These observations show that the LD mean speed and mean travel distances are affected only upon direct binding to DII-core^G161F^.

### Lower Frequency of High Velocity Travel Runs and High Frequency of Pauses Contribute to Slower Mean Speeds for DII-core Coated LDs

**Figure 4:**
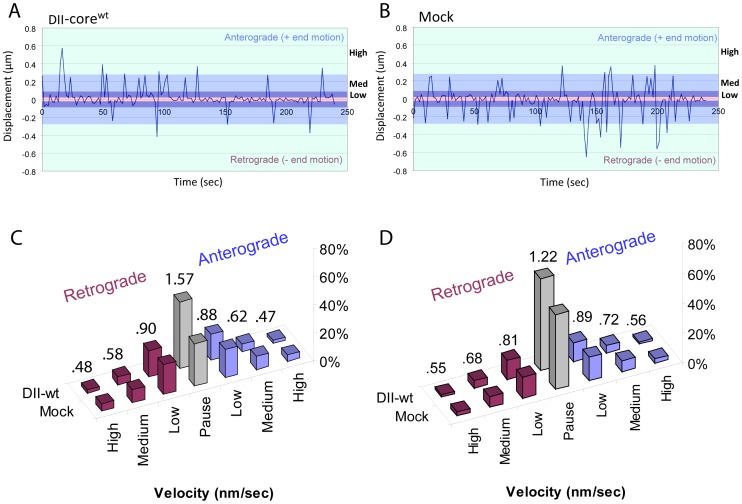
DII-core^wt^ coated LD velocities measured in naïve Huh-7 cells and Huh-7 cells stably expressing an HCV subgenomic replicon. (A–B) Average representative measurement of a much larger data set, LD velocities in retrograde or anterograde directed transport are measured in Huh-7 cells expressing (A) DII-core^wt^ and (B) mock transfected. The velocity amplitudes at each time point are divided into parameters of, low, medium, and high velocities for both directions. The pink parameter line is indicated by a paused event, which was determined by obtaining the average speed of LDs from nocadazole treated Huh-7 cells. (C–D) The frequency of low (15.7 nm/sec –50 nm/sec), medium (50.1 nm/sec –180 nm/sec), and high velocity (>180.1 nm/sec) measurements, expressed as a percentage, in both directions, are plotted after particle tracking LDs in DII-core^wt^ expressing (C) Huh-7 cells, and (D) Huh-7 cells harbouring an HCV subgenomic replicon. The velocities are measured for DII-core^wt^ coated LDs in DII-core^wt^ expressing cells, and LDs from mock cells not expressing DII-core^wt^. The ratios above each set of columns are calculated by dividing the frequency for each velocity interval of DII-core^wt^ coated LDs by their respective mock LDs.

To investigate DII-core’s induced suppression of the mean LD speed, we explored the frequency of low to high instantaneous velocities of DII-core^wt^ coated LDs compared to LDs in mock cells. Since dynein and kinesin motors mediate cargo transport in opposite directions, measured LD velocities can provide information about whether the mobility of DII-core^wt^ coated LDs travel more frequently towards one direction, and thus, reveal differential activity between the two motors. The trajectories of individual LDs were tracked using the center of the nucleus as a fixed point relative to the position of the LD. LD travel runs that were directed towards the MTOC (retrograde manner) were identified as negative displacement, while LDs that moved away from the MTOC (anterograde motion), were identified as having positive displacement (Figure 4A–B). The differential velocity profiles were then segregated into low (15.7–50 nm/sec), medium (50–180 nm/sec), and high velocity (>180 nm/sec) travel runs (Figure 4C–D). LD particle tracking in both directions revealed that the frequency of high and medium velocity travel runs for DII-core^wt^ coated LDs was lower when compared to LDs from the mock sample (Figure 4C). This is represented as a ratio for the frequency for DII-core^wt^ coated LDs divided by LDs from the mock, with similar ratios determined for both directions. For example, at high velocity travel runs, the ratios were calculated to be 0.47 for the anterograde direction, and 0.48 for the retrograde direction (Figure 4C). The differential frequencies for the high and medium velocities were also consistent with DII-core^wt^ coated LDs in Huh-7 cells expressing a subgenomic replicon of HCV (Figure 4D). Therefore, the shorter travel distances of DII-core^wt^ coated LDs is reflected in the lower frequency of high velocity travel runs, and is independent of the presence of non-structural HCV proteins that are involved in membranous web formation and viral replication.

Next, we investigated the differential velocity profiles for the DII-core^161^ mutant coated LDs to determine if binding strength is reflected in the frequency of high velocity travel runs. As shown in Figure S3, high velocity travel runs were less frequent for DII-core^161^ mutant coated LDs, with relative differences in ratios corresponding to their expected LD binding strength. This was clear for the highest binding strength mutant DII-core^G161F^, with ratios of 0.32 and 0.31 for retrograde and anterograde velocities, respectively (Figure S3A). Reduced strength of binding to LDs, as seen with the DII-core^G161S^ mutant, demonstrated the highest ratios of 0.95 and 0.83 for retrograde and anterograde velocities, respectively (Figure S3D). Overall, distances traveled by mutant DII-core^161^ coated LDs appear to correlate with relative frequency of high velocity LD travel runs.

While observing LD transport, we found that not only do LDs travel at various velocities, but they also appear to pause in a stalled state. We postulated that higher frequencies of LD pauses can also contribute to smaller mean distances traveled by DII-core coated LDs. To establish the minimal threshold that would identify LD movement by active transport, we used a microtubule depolymerizing drug, nocadazole, which halts motor protein dependent active transport. We have previously reported that LDs in cells treated with nocadazole were found to move at approximately 15.7 nm/sec [17]; velocities below this threshold were characterized as pauses (Figure 4, Figure S3), and LD movement above this speed was placed in a range of low, medium, or high velocity travel runs. In general, pauses were more frequent for all DII-core^161^ mutants and DII-core^wt^ coated LDs compared to mock controls (Figure S3A–D). Since LDs are often observed to move back and forth in opposite directions, we further calculated the frequency of directional switches. We observed no clear trend that correlated directional switches with LD binding strength (data not shown). Therefore, the higher pause frequency for DII-core coated LDs, and not frequency of directional switching, is likely to contribute to the shortening of mean LD travel distances and mean LD speed.

### DII-core bound LDs Spend Equal amounts of Time Traveling in both the Retrograde and Anterograde Direction

**Figure 5:**
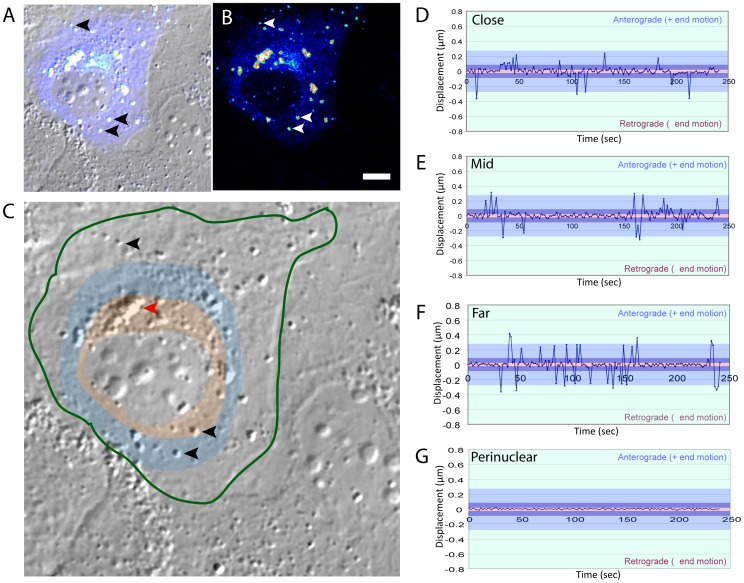
Tracking LD mobility at distinct locations of the cell. While all of the mutants were tracked accordingly, Huh-7 cells expressing DII-core^G161A^ is a representative image acquired from a large data set. Huh-7 cells expressing DII-core^G161A^ is shown as (A) a merged image of DIC and TPF, and (B) TPF. DII-core^G161A^ coated LDs are selected, and indicated by the arrows, to demonstrate fluorescence overlap between TPF and DIC. (C) LDs localized at different areas within the transfected cell (green outline) were segregated into regions relative to the center of the nucleus, such as close (orange shading), mid (blue), and far (no shading). Each black arrow represents a DII-core^G161A^ coated LD for each of the segregated region, and the velocities were measured for each direction in the close (D), mid (E), far (F) regions. The red arrow selects for a region of dense LDs in the perinuclear region with higher levels of DII-core^G161A^. (G) The velocity of the LD, identified by the red arrow was measured. All scale bars represent 10 µm.

We have previously used live-cell imaging by CARS and DIC microscopy to visualize the ability of full-length core protein to induce LD migration towards the perinuclear region associated with HCV replication and assembly [17]. Based on these data and published work by Boulant *et al.*, it was suggested that core may directly or indirectly favor a molecular motor imbalance by perturbing the mechanics of one motor over the other [35]. Since expression of full-length and DII-core induces LD migration towards the perinuclear region, a molecular motor imbalance should drive a greater frequency of travel runs in the retrograde direction. For this reason, we counted the total frequency of travel runs for one direction that combined low, medium, and high velocity travel runs. However, the frequency of travel runs for wt and mutant DII-core coated LDs were similar in both directions over our four minute time course (Figure S3E). Finally, directionality of LD travel was assessed against cytoplasmic location, relative to the nucleus, since DII-core coated LDs were also observed to be scattered throughout the cell (Figure S4). Cells were divided into regions, as shown in Figure 5C, with regions identified as close to the perinuclear region (close), middle of the cytoplasm (mid), and in the cell periphery (far). However, a trend was not observed for wild-type and mutant DII-core coated LD velocities. This suggests that at time of analysis, movement of DII-core coated LDs travel equally in both directions and is unrelated to its location in the cell, except when the LDs reach the perinuclear region. Although our time measurements last approximately four minutes, we have included a large data set and statistics measured from all regions of the cell. Importantly, we wanted to measure the movement of LDs at a particular stage during core expression, before core induces LD accumulation in the perinuclear region. While it is difficult to normalize our data to 48–72 hours during the time span of infection, LD mobility measurements required video-rate imaging that is attainable over a shorter time course with averaging of many trials.

### LDs at the Extreme Perinuclear Region Demonstrate Limited Mobility

Two general localizations of DII-core coated LDs were revealed by CARS and DIC imaging for wild-type and all the DII-core mutants: scattered throughout the cell (Figure S5, white arrowheads), and tightly aggregated in the perinuclear region (Figure S5, red arrowheads). Perinuclear LD aggregation was not observed in mock cells, where LDs were generally observed to be scattered throughout the entire cell. Up until this point, particle tracking was focused on DII-core coated LDs scattered throughout the cell to investigate dynamics of LD mobility resulting from differential binding strengths of DII-core. Therefore, using DII-core^G161A^ as a representative image, we measured the velocities of the perinuclear aggregates (Figure 5G). We found that these large LD aggregates were tightly localized together and had limited mobility (Figure 5C&G). In contrast, DII-core coated LDs outside of this region experienced bidirectional travel runs (Figure 5D–F). These observations suggest that, although DII-core coated LDs remain mobile at areas outside the perinuclear region, mobility is abrogated once they reach the perinuclear region.

## Discussion

Molecular motors, such as dynein and kinesin, function by a mechanoenzyme core containing ATPase activity that facilitates active transport along the plus-end (towards cell periphery) and minus-end (towards nucleus) of microtubules [15], [57]. Upon entry, viruses are capable of binding to molecular motor proteins that move on microtubules to achieve transport. This is common for adenovirus, herpes simplex virus (HSV), and human immunodeficiency virus (HIV), which have capsid and tegument viral proteins on the surface of their viral particles that, upon entry into the cell, bind and travel by motor-induced transport to ensure that viral particles are properly delivered to specific cellular regions to establish infection [58], [59], [60], [61], [62]. Likewise, HCV is able to directly use the microtubule network upon cell entry [63], as well as indirectly after viral RNA translation whereby viral proteins are the likely components that mediate interactions with motor proteins [37]. As such, the binding of HCV core protein to LDs is critical in manipulating LD transport towards the perinuclear region, and is required in the early stages of viral assembly [35]. However, our understanding of core-induced modulation of dynamic LD trafficking is limited.

### DII-core Coated LD Dynamics

LDs migrate towards the perinuclear region as early as 20 hours post-expression of core protein [17], [35]. Based on this evidence, we aimed to capture the dynamics of LD motions just prior to this time point at a pertinent stage when LDs are targeted by DII-core for viral induced trafficking. In this study, molecular imaging was used to track LD trajectories in live hepatocytes in the presence and absence of bound DII-core protein, as well as bound mutant DII-core having variable binding strength to LDs. As seen with full-length core [17], expression of wt and G161 mutant GFP-tagged DII-core protein colocalized with LDs and induced LD migration to the perinuclear region (Figure 1D and Figure 3A–D). Supporting earlier evidence of full-length core’s ability to modulate LD dynamics [17], LDs coated with wt and mutant DII-core showed slower mean speeds and a decrease in mean travel distances (Table 1). In this work, the ability to directly compare naïve and GFP-tagged DII-core coated LDs was imperative in understanding finer details LD dynamics, such as decreased high velocity travel runs and more pauses for DII-core coated LDs compared to LDs in cells not expressing DII-core (Figure 4 and Figure S3).

Mutations in DII-core that impact protein structure and/or LD affinity influence viral assembly and, hence, virion production [44], [45], [64]. Since core mobility, as reflected in its ability to associate and be released from the LD, appears to be important for virion assembly [45], we investigated mobility of LDs bound to DII-core containing mutations at a glycine residue predicted to lie at the membrane interface [55]. We have found that mutations at G161 impacted DII-core LD binding strength with respect to the hydrophobicity of the residue (Filipe *et al*., manuscript in preparation). Similarly, our results revealed a trend in LD mobility ranging from slower speeds and decreased travel distances when the LD binding strength of DII-core is increased (Table 1). This trend of reduced mobility upon increased LD binding appears to follow the whole residue free energies determined for the transfer from water to a unilamellar vesicle interface (reported in [65]). Furthermore, distances traveled by mutant DII-core^161^ coated LDs correlated with relative frequency of high velocity LD travel runs. These results suggest that the greater the time DII-core protein spends on the LD surface, the greater LD dynamics deviate from normal. Consistent with this, Counihan *et al.* also observed that core coated LDs decreased in their motility [66]. Previous mutations in this region of core have been shown to be critical in viral particle assembly [64]. Our results suggest that core’s LD binding strength and effect on LD speed may play a role in virion assembly.

### Bidirectional Movement of DII-core Coated LDs

Current models propose that both dynein and kinesin remain associated with cargo during transport, even if only one motor is active [12], [16], [67]. Our data supports bidirectional movement for both naïve and DII-core coated LDs, which confirms that both molecular motors remain bound and functional (Figure 4 and Figure S3). Bidirectional movement was observed for both wt and mutant DII-core coated LDs, which suggests that overall directional movement initiated by core is likely not based on LD binding strength. Furthermore, the prevalence of retrograde and anterograde directed transport of DII-core coated LDs was equally observed (Figure S3E). Since we showed localization of LDs to the perinuclear region, as well as equal bidirectional movements, it is unlikely that motor imbalance is the sole cause for core-mediated LD localization to the perinuclear region of the cell. It is possible that our imaging experiments over a four minute time course was too short to adequately capture an imbalance between active motors. However, since we observed many LDs at different locations within the cell, it is possible that perinuclear localization is driven by detachment from microtubules at the destination rather than a dramatic change in motor protein function.

Perinuclear localization and bidirectional movement is not limited to core-bound LDs, since it has been shown for other pathogens as well. For example, Suomalainen *et al.* demonstrated that despite observation of bidirectional motions, overall net movement of newly entered adenovirus particles was directed towards the perinuclear region [68]. The authors showed that localization to the perinuclear region was dependent on transient activation of protein kinase A (PKA) and the p38/mitogen-activated protein kinase (MAPK) pathway. Similar mechanisms may be involved in core protein induced perinuclear localization and bidirectional movement of LDs, since core expression has been shown to activate the p38/MAPK pathway in hepatocytes [69], [70], [71].

### DII-core Limits LD Mobility within the Perinuclear Region

We highlighted important dynamics of core-directed mobility for DII-core coated LDs, and demonstrated that bidirectional motion is observed for all other DII-core coated LDs that are located outside of the critical perinuclear region (Figure 5). This prompted us to investigate the movement of LDs within perinuclear regions that also have higher levels of localized DII-core protein (Figure 5C & G). Our results revealed minimal LD movement, indicative of exclusively paused or trapped LDs. The ability of DII-core to limit LD mobility within the perinuclear region could be the result of molecular motor disengagement, allowing DII-core coated LDs to accumulate. Indeed, Miyanari *et al.* reported that LDs are required at the replication and assembly sites [49], where LD accumulation could effectively link early and late viral assembly stages.

Alternatively, DII-core coated LDs may be stabilized in the perinuclear region by the recruitment of additional host proteins. One method of stability could be mediated through hijacking the autophagic pathway that forms aggresomes within sites of replication and assembly [72], [73]. The accumulation of aggresomes sequestered around the MTOC could prevent motors from binding and result in densely packed LDs that are stabilized without accessible motor proteins. Alternatively, host proteins that are recruited to LDs by core protein at the perinuclear regions may play a role in stabilizing LDs at these sites. Recently, a subunit of host clathrin adaptor protein complex 2 (AP2M1) has been shown to bind core by recognizing a highly conserved tyrosine-based sorting signal along the DII region [74]. These proteins sort intracellular cargo via a clathrin adaptor and can mediate endocytic functions. Importantly, interference of the AP2M1-core interaction prevents viral assembly [74]. The same AP2M1 binding motif was also identified in the region of the viral E1 glycoprotein. Such a feature supports the idea that AP2M1 is recruited to LDs via DII of core protein, and likely mediates intracellular trafficking of core to sites of assembly where E1 and E2 proteins reside, prior to the envelopment of the viral particle. This engagement can possibly stabilize the LD from being bound to motor proteins once LDs reach this critical area at the perinuclear region.

Aggregation of DII-core coated LDs in the perinuclear region may also result in disconnection from normal metabolic processes. The increased LD volume detected in the periphery of wt and mutant DII-core expressing cells is consistent with a reduction in core-induced TG turnover in LDs, opposed to TG synthesis, as previously determined by Harris *et al* [75]. Based on our identification of restricted LD mobility of perinuclear aggregates, it is tempting to hypothesize that this mechanism of aggregation could further limit normal lipid turnover. We have also recently shown that DII-core is sufficient to initiate a significant change in NAD(P)H levels, as measured by fluorescence lifetime imaging, which may also influence LD biogenesis and localization (71).

## Conclusions

In this study, we have revealed insight into HCV core protein’s dynamic control of LD migration. We showed that DII-core causes a decrease in LD speed similar to full-length core, with limited effect on directionality within the time frame of our experiments. We also found that the binding strength of DII-core further impacted LD mobility, indicating that DII-core has a temporal impact on the LD with respect to time associated with the LD. Moreover, the observed bidirectional transport of DII-core coated LDs may suggest that additional host proteins are essential in directing transport of core-coated LDs, and these potential interactions will be the focus of future studies. Nevertheless, live-cell imaging has revealed several novel aspects of core-induced LD mobility.

## Materials and Methods

### Tissue Culture

Human hepatoma cells (Huh-7) were grown in DMEM medium supplemented with 100 nM nonessential amino acids, 50 U/mL penicillin, 50 µg/mL streptomycin, and 10% FBS (CANSERA, Rexdale,ON). Huh-7 cells harboring the pFK-I389neo/NS3-3′/5.1 subgenomic replicon were maintained in the same culture medium supplemented with 250 µg/mL G418 Geneticin (GIBCO-BRL, Burlington, ON). The pFK-I389neo/NS3-3′/5.1 subgenomic replicon was kindly provided by Ralf Bartenschlager (University of Heidelberg, Germany).

### Overexpression of HCV DII-core Protein

Huh-7 cells were seeded at 8.0×10^4^ cells/well in borosilicate Lab-Tek chambers (VWR, Mississauga, ON). After 24 h, at a confluency of 60–70%, cells were transfected with plasmids that expressed wild-type (wt) and mutant DII-core suspended in transfection media including lipofectamine 2000 (Invitrogen Canada Inc., Burlington, ON). After 4 h, DMEM in 20% FBS was added in equal volume to the chambers. Details of the GFP-tagged DII-core^wt^ construct are described elsewhere [47]. QuikChange site-directed mutagenesis (Stratagene) and primer design were performed according to the manufacturer’s guidelines, and confirmed by sequencing.

### Coherent Anti-Stokes Raman Scattering, Two-photon Fluorescence and Differential Interference Contrast Microscopies

The CARS microscopy system employed a single femtosecond Ti:sapphire oscillator as the excitation source, as previously described [51], [52]. An Olympus FV300 laser scanning microscopy system on an IX71 inverted microscope was utilised for imaging experiments. A 40x Uapo 1.15NA water immersion objective and a long working distance 0.55 NA condenser were used. The FV300 was adapted for TPF. Source was a Coherent Mira 900 Ti:sapphire laser producing pulses of approximately 100 fs at 800 nm wavelength with an 80 MHz repetition rate. Laser scanning microscopy can be readily adapted to DIC by taking advantage of the high inherent polarization in most laser sources. The DIC optics were adjusted as they would typically be for transmitted light use: with the prisms removed the condenser polarizer was adjusted to cross with the objective polarizer. For laser scanning, the analyzer, which is in a fluorescence cube in the IX71, was removed from the beam path. To optimally align the polarization of the laser with that of the microscope optics, a 700–1000 nm achromatic half wave plate (WPA1212 Casix) was placed in the laser path before entering the FV300 scan-box. The polarization of the laser was adjusted by rotating this wave plate to minimize the amount of light collected through the condenser polarizer. The DIC prisms were inserted and the path and the bias of the objective prism adjusted to the optimal image.

### Particle Tracking of LDs in Huh-7 Cells

Particle tracking of LD motion for both speed and distance was captured using spot tracker add-on with ImageJ, as previously described [17]. The spot tracker followed the light shaded halo contrast of LDs as a result of changes in refractive index captured by DIC imaging. The measurement of directional motion was calculated from a fixed reference point in the center of the nucleus relative to the LD at each frame.

### Quantitative Voxel Analysis

Quantitative data from the CARS images was determined using a voxel counting routine in ImageJ as previously described [17], [76], [77]. In each image, multiple cells within the same field of view were counted to generate an average percentage of lipid volume.

## Supporting Information

Figure S1 **Particle tracking DII-core^wt^ coated LDs in Huh-7 cells stably expressing an HCV subgenomic replicon.** (A) CARS and TPF microscopy captures colocalization between DII-core^wt^ and LDs, and captures DII-core^wt^-induced LD localization at the perinuclear region. Panel 4 is a magnified image selected by a region of interest from the merged image to project a clearer view of colocalization between DII-core^wt^ and LDs. (B) Particle tracking DII-core^wt^ coated LDs and LDs in mock cells not expressing DII-core^wt^. The overall mean travel distance and mean speeds were measured. The ratio is calculated by dividing the mean speed of DII-core^wt^ coated LDs by LDs from the mock sample. The n represents the number of LDs that were particle tracked. Live-cell imaging was conducted for duration of four minutes with each frame interval acquired at 1.65 sec/frame. All scale bars represent 10 µm.
(TIF)

Figure S2 **DII-core^G161F^ coated LDs are particle tracked using simultaneous TPF and DIC microscopy.** This is a representative image of DII-core^G161F^ expressed in Huh-7 cells. Three individual LDs with dissimilar environments were selected (A–C, white arrows), and their trajectories were measured to calculate the overall distances traveled. (D) A larger DIC image of (B) includes boxes to identify each LD trajectory (inset 1–3). The value above each box (D) indicates their overall travel distances for (1) DII-core^G161F^ coated LD (2) non DII-core^G161F^ coated LD within the same cell, (3) and a LD in an adjacent cell not expressing DII-core^G161F^. Each LD trajectory is magnified to demonstrate the LD track with selective freeze frame time-intervals representing the LD position at their indicated times. Due to frequent bidirectional movements, the displayed trajectories represent a general movement path, and does not portray total distance. All of the LDs are tracked according to the same start and end time. All scale bars represent 10 µm.
(TIF)

Figure S3 **LD velocities are measured in Huh-7 cells expressing DII-core^161^ mutants.** (A–D) The frequency of pauses (<15.7 nm/sec), low (15.7 nm/sec –50 nm/sec), medium (50.1 nm/sec –180 nm/sec), and high velocity (>180.1 nm/sec) measurements, expressed as a percentage, in both directions are plotted for LDs in cells expressing (A) DII-core^G161F^, (B) DII-core^G161L^, (C) DII-core^G161A^, (D) DII-core^G161S^. The ratios above each set of columns is calculated by dividing the frequency for each velocity interval of DII-core coated LDs by their respective mock LDs. (E) The total frequency of retrograde, anterograde, and pauses were also collected and presented as a fold-change measurement that compared LDs in all DII-core^161^ mutants with each of their respective mocks.
(TIF)

Figure S4 **Frequency of LD velocities at three different regions in Huh-7 cells expressing DII-core^mut^ and in the mock.** The average frequency of low, medium, and high velocity runs for each direction was calculated for LDs bound to (A) DII-core^wt^, (B) DII-core^G161F^, (C) DII-core^G161L^, (D) DII-core^G161S^, (E) DII-core^G161A^. The data was separated according to where the LD was located at a position that was relative to the nucleus, either at a close, medium, or far location.
(TIF)

Figure S5 **Two populations of DII-core^G161A^ coated LDs was observed.** This is a representative image with a pattern that is typically observed in all other DII-core constructs. The white arrow represents a LD population of individual LDs that are bound to DII-core^G161A^. The red arrow corresponds to tightly packed LDs with a high abundance of DII-core^G161A^ colocalized at the same region. Individual LDs are indistinguishable at this region. All scale bars represent 10 µm.
(TIF)

## References

[1] Farese RV Jr, Walther TC (2009) Lipid droplets finally get a little R-E-S-P-E-C-T. Cell 139: 855–860. 19945371 10.1016/j.cell.2009.11.005 PMC3097139

[2] Martin S, Parton RG (2006) Lipid droplets: a unified view of a dynamic organelle. Nat Rev Mol Cell Biol 7: 373–378. 16550215 10.1038/nrm1912

[3] Walther TC, Farese RV Jr (2009) The life of lipid droplets. Biochim Biophys Acta 1791: 459–466. 19041421 10.1016/j.bbalip.2008.10.009 PMC2782899

[4] Murphy DJ (2001) The biogenesis and functions of lipid bodies in animals, plants and microorganisms. Prog Lipid Res 40: 325–438. 11470496 10.1016/s0163-7827(01)00013-3

[5] Bickel PE, Tansey JT, Welte MA (2009) PAT proteins, an ancient family of lipid droplet proteins that regulate cellular lipid stores. Biochim Biophys Acta 1791: 419–440. 19375517 10.1016/j.bbalip.2009.04.002 PMC2782626

[6] Brasaemle DL (2007) Thematic review series: adipocyte biology. The perilipin family of structural lipid droplet proteins: stabilization of lipid droplets and control of lipolysis. J Lipid Res 48: 2547–2559. 17878492 10.1194/jlr.R700014-JLR200

[7] Walther TC, Farese RV Jr (2012) Lipid droplets and cellular lipid metabolism. Annu Rev Biochem 81: 687–714. 22524315 10.1146/annurev-biochem-061009-102430 PMC3767414

[8] Bostrom P, Rutberg M, Ericsson J, Holmdahl P, Andersson L, et al (2005) Cytosolic lipid droplets increase in size by microtubule-dependent complex formation. Arterioscler Thromb Vasc Biol 25: 1945–1951. 16051877 10.1161/01.ATV.0000179676.41064.d4

[9] Kunwar A, Tripathy S, Xu J, Mattson M, Anand P, et al (2011) Mechanical stochastic tug-of-war models cannot explain bidirectional lipid-droplet transport. Proc Natl Acad Sci USA 108: 18960–18965. 22084076 10.1073/pnas.1107841108 PMC3223464

[10] Targett-Adams P, Chambers D, Gledhill S, Hope RG, Coy JF, et al (2003) Live cell analysis and targeting of the lipid droplet-binding adipocyte differentiation-related protein. J Biol Chem 278: 15998–16007. 12591929 10.1074/jbc.M211289200

[11] Welte MA, Gross SP, Postner M, Block SM, Wieschaus EF (1998) Developmental regulation of vesicle transport in Drosophila embryos: forces and kinetics. Cell 92: 547–557. 9491895 10.1016/s0092-8674(00)80947-2

[12] Gross S, Vershinin M, Shubeita G (2007) Cargo transport: two motors are sometimes better than one. Curr Biol 17: R478–486. 17580082 10.1016/j.cub.2007.04.025

[13] Gross SP, Welte MA, Block SM, Wieschaus EF (2000) Dynein-mediated cargo transport in vivo. A switch controls travel distance. J Cell Biol 148: 945–956. 10704445 10.1083/jcb.148.5.945 PMC2174539

[14] Kulic I, Brown A, Kim H, Kural C, Blehm B, et al (2008) The role of microtubule movement in bidirectional organelle transport. Proc Natl Acad Sci USA 105: 10011–10017. 18626022 10.1073/pnas.0800031105 PMC2481308

[15] Welte M (2004) Bidirectional transport along microtubules. Curr Biol 14: R525–537. 15242636 10.1016/j.cub.2004.06.045

[16] Kural C, Kim H, Syed S, Goshima G, Gelfand VI, et al (2005) Kinesin and Dynein Move a Peroxisome in Vivo: A Tug-of-War or Coordinated Movement? Science 308: 1469–1472. 15817813 10.1126/science.1108408

[17] Lyn RK, Kennedy DC, Stolow A, Ridsdale A, Pezacki JP (2010) Dynamics of lipid droplets induced by the hepatitis C virus core protein. Biochem Biophys Res Commun 399: 518–542. 20678475 10.1016/j.bbrc.2010.07.101

[18] Muller MJ, Klumpp S, Lipowsky R (2008) Tug-of-war as a cooperative mechanism for bidirectional cargo transport by molecular motors. Proc Natl Acad Sci USA 105: 4609–4614. 18347340 10.1073/pnas.0706825105 PMC2290779

[19] Herker E, Ott M (2012) Emerging role of lipid droplets in host/pathogen interactions. J Biol Chem 287: 2280–2287. 22090026 10.1074/jbc.R111.300202 PMC3268388

[20] Pezacki JP, Singaravelu R, Lyn RK (2010) Host-virus interactions during hepatitis C virus infection: a complex and dynamic molecular biosystem. Mol Biosyst 6: 1131–1142. 20549003 10.1039/b924668c

[21] Lavanchy D (2011) Evolving epidemiology of hepatitis C virus. Clin Microbiol Infect 17: 107–115. 21091831 10.1111/j.1469-0691.2010.03432.x

[22] Tang H, Grise H (2009) Cellular and molecular biology of HCV infection and hepatitis. Clin Sci 117: 49–65. 19515018 10.1042/CS20080631

[23] Alvisi G, Madan V, Bartenschlager R (2011) Hepatitis c virus and host cell lipids: An intimate connection. RNA Biol 8: 258–269. 21593584 10.4161/rna.8.2.15011

[24] Herker E, Harris C, Hernandez Cl, Carpentier A, Kaehlcke K, et al (2010) Efficient hepatitis C virus particle formation requires diacylglycerol acyltransferase-1. Nat Med 16: 1295–1303. 20935628 10.1038/nm.2238 PMC3431199

[25] Syed G, Amako Y, Siddiqui A (2010) Hepatitis C virus hijacks host lipid metabolism. Trends Endocrinol Metab 21: 33–73. 19854061 10.1016/j.tem.2009.07.005 PMC2818172

[26] Chisari F (2005) Unscrambling hepatitis C virus-host interactions. Nature 436: 930–932. 16107831 10.1038/nature04076

[27] Clément S, Negro F (2007) Hepatitis C virus: the viral way to fatty liver. J Hepatol 46: 985–992. 17445938 10.1016/j.jhep.2007.03.005

[28] McLauchlan J (2009) Lipid droplets and hepatitis C virus infection. Biochim Biophys Acta 1791: 552–561. 19167518 10.1016/j.bbalip.2008.12.012

[29] Saka HA, Valdivia R (2012) Emerging Roles for Lipid Droplets in Immunity and Host-Pathogen Interactions. Annu Rev Cell Dev Biol 28: 411–437. 22578141 10.1146/annurev-cellbio-092910-153958

[30] Su A, Pezacki J, Wodicka L, Brideau A, Supekova L, et al (2002) Genomic analysis of the host response to hepatitis C virus infection. Proc Natl Acad Sci USA 99: 15669–15743. 12441396 10.1073/pnas.202608199 PMC137774

[31] Jackel-Cram C, Babiuk LA, Liu Q (2007) Up-regulation of fatty acid synthase promoter by hepatitis C virus core protein: genotype-3a core has a stronger effect than genotype-1b core. J Hepatol 46: 999–1008. 17188392 10.1016/j.jhep.2006.10.019

[32] Roingeard P, Depla M (2011) The birth and life of lipid droplets: learning from the hepatitis C virus. Biol Cell 103: 223–231. 21488839 10.1042/BC20100119

[33] Moradpour D, Penin Fo, Rice C (2007) Replication of hepatitis C virus. Nat Rev Microbiol 5: 453–463. 17487147 10.1038/nrmicro1645

[34] Barba G, Harper F, Harada T, Kohara M, Goulinet S, et al (1997) Hepatitis C virus core protein shows a cytoplasmic localization and associates to cellular lipid storage droplets. Proc Natl Acad Sci USA 94: 1200–1205. 9037030 10.1073/pnas.94.4.1200 PMC19768

[35] Boulant S, Douglas M, Moody L, Budkowska A, Targett-Adams P, et al (2008) Hepatitis C virus core protein induces lipid droplet redistribution in a microtubule- and dynein-dependent manner. Traffic 9: 1268–1350. 18489704 10.1111/j.1600-0854.2008.00767.x

[36] Ait-Goughoulte M, Hourioux C, Patient R, Trassard S, Brand D, et al (2006) Core protein cleavage by signal peptide peptidase is required for hepatitis C virus-like particle assembly. J Gen Virol 87: 855–915. 16528035 10.1099/vir.0.81664-0 PMC2220033

[37] McLauchlan J, Lemberg MK, Hope G, Martoglio B (2002) Intramembrane proteolysis promotes trafficking of hepatitis C virus core protein to lipid droplets. The EMBO J 21: 3980–3988. 12145199 10.1093/emboj/cdf414 PMC126158

[38] Okamoto K, Mori Y, Komoda Y, Okamoto T, Okochi M, et al (2008) Intramembrane processing by signal peptide peptidase regulates the membrane localization of hepatitis C virus core protein and viral propagation. J Virol 82: 8349–8410. 18562515 10.1128/JVI.00306-08 PMC2519675

[39] Perez-Berna AJ, Veiga AS, Castanho MA, Villalain J (2008) Hepatitis C virus core protein binding to lipid membranes: the role of domains 1 and 2. J Viral Hepat 15: 346–402. 18179451 10.1111/j.1365-2893.2007.00948.x PMC7166730

[40] Depla M, Uzbekov R, Hourioux C, Blanchard E, Le Gouge A, et al (2010) Ultrastructural and quantitative analysis of the lipid droplet clustering induced by hepatitis C virus core protein. Cell Mol Life Sci 67: 3151–3161. 20422251 10.1007/s00018-010-0373-z PMC11115826

[41] Kopp M, Murray C, Jones C, Rice C (2010) Genetic analysis of the carboxy-terminal region of the hepatitis C virus core protein. J Virol 84: 1666–1739. 20007277 10.1128/JVI.02043-09 PMC2812405

[42] Targett-Adams P, Hope G, Boulant S, McLauchlan J (2008) Maturation of hepatitis C virus core protein by signal peptide peptidase is required for virus production. J Biol Chem 283: 16850–16859. 18424431 10.1074/jbc.M802273200

[43] Alsaleh K, Delavalle P-Y, Pillez A, Duverlie G, Descamps V, et al (2010) Identification of basic amino acids at the N-terminal end of the core protein that are crucial for hepatitis C virus infectivity. J Virol 84: 12515–12543. 20943968 10.1128/JVI.01393-10 PMC3004332

[44] Boulant S, Targett-Adams P, McLauchlan J (2007) Disrupting the association of hepatitis C virus core protein with lipid droplets correlates with a loss in production of infectious virus. J Gen Virol 88: 2204–2217. 17622624 10.1099/vir.0.82898-0

[45] Shavinskaya A, Boulant S, Penin F, McLauchlan J, Bartenschlager R (2007) The lipid droplet binding domain of hepatitis C virus core protein is a major determinant for efficient virus assembly. J Biol Chem 282: 37158–37169. 17942391 10.1074/jbc.M707329200

[46] Jones D, McLauchlan J (2010) Hepatitis C virus: assembly and release of virus particles. J Biol Chem 285: 22733–22742. 20457608 10.1074/jbc.R110.133017 PMC2906262

[47] Lai C-K, Jeng K-S, Machida K, Lai M (2010) Hepatitis C virus egress and release depend on endosomal trafficking of core protein. J Virol 84: 11590–11598. 20739534 10.1128/JVI.00587-10 PMC2953145

[48] Wang C, Gale M Jr, Keller BC, Huang H, Brown MS, et al (2005) Identification of FBL2 as a geranylgeranylated cellular protein required for hepatitis C virus RNA replication. Mol Cell 18: 425–434. 15893726 10.1016/j.molcel.2005.04.004

[49] Miyanari Y, Atsuzawa K, Usuda N, Watashi K, Hishiki T, et al (2007) The lipid droplet is an important organelle for hepatitis C virus production. Nat Cell Biol 9: 1089–1186. 17721513 10.1038/ncb1631

[50] Evans CL, Potma EO, Puoris’haag M, Côté D, Lin CP, et al (2005) Chemical imaging of tissue in vivo with video-rate coherent anti-Stokes Raman scattering microscopy. Proc Natl Acad Sci USA 102: 16807–16812. 16263923 10.1073/pnas.0508282102 PMC1283840

[51] Pegoraro AF, Ridsdale A, Moffatt DJ, Jia Y, Pezacki JP, et al (2009) Optimally chirped multimodal CARS microscopy based on a single Ti:sapphire oscillator. Opt Express 17: 2984–2996. 19219203 10.1364/oe.17.002984

[52] Pegoraro AF, Ridsdale A, Moffatt DJ, Pezacki JP, Thomas B, et al (2009) All-fiber CARS microscopy of live cells. Opt Express 17: 20700–20706. 19997300 10.1364/OE.17.020700

[53] Pezacki JP, Blake JA, Danielson D, Kennedy DC, Lyn RK, et al (2011) Chemical contrast for imaging living systems: molecular vibrations drive CARS microscopy. Nat Chem Biol 7: 137–182. 21321552 10.1038/nchembio.525 PMC7098185

[54] Egger D, Wolk B, Gosert R, Bianchi L, Blum HE, et al (2002) Expression of hepatitis C virus proteins induces distinct membrane alterations including a candidate viral replication complex. J Virol 76: 5974–5984. 12021330 10.1128/JVI.76.12.5974-5984.2002 PMC136238

[55] Boulant S, Montserret R, Hope R, Ratinier M, Targett-Adams P, et al (2006) Structural determinants that target the hepatitis C virus core protein to lipid droplets. J Biol Chem 281: 22236–22247. 16704979 10.1074/jbc.M601031200

[56] Boulant S, Vanbelle C, Ebel C, Penin F, Lavergne J-P (2005) Hepatitis C Virus Core Protein Is a Dimeric Alpha-Helical Protein Exhibiting Membrane Protein Features. J Virol 79: 11353–11365. 16103187 10.1128/JVI.79.17.11353-11365.2005 PMC1193582

[57] Burgess SA, Walker ML, Sakakibara H, Knight PJ, Oiwa K (2003) Dynein structure and power stroke. Nature 421: 715–718. 12610617 10.1038/nature01377

[58] Dodding M, Way M (2011) Coupling viruses to dynein and kinesin-1. EMBO J 30: 3527–3566. 21878994 10.1038/emboj.2011.283 PMC3181490

[59] Henry T, Gorvel J-P, Méresse S (2006) Molecular motors hijacking by intracellular pathogens. Cell Microbiol 8: 23–32. 16367863 10.1111/j.1462-5822.2005.00649.x

[60] Lyman MG, Enquist LW (2009) Herpesvirus Interactions with the Host Cytoskeleton. J Virol 83: 2058–2066. 18842724 10.1128/JVI.01718-08 PMC2643721

[61] McDonald D, Vodicka MA, Lucero G, Svitkina TM, Borisy GG, et al (2002) Visualization of the intracellular behavior of HIV in living cells. J Cell Biol 159: 441–452. 12417576 10.1083/jcb.200203150 PMC2173076

[62] Suomalainen M, Nakano MY, Keller S, Boucke K, Stidwill RP, et al (1999) Microtubule-dependent plus- and minus end-directed motilities are competing processes for nuclear targeting of adenovirus. J Cell Biol 144: 657–672. 10037788 10.1083/jcb.144.4.657 PMC2132937

[63] Roohvand F, Maillard P, Lavergne JP, Boulant S, Walic M, et al (2009) Initiation of hepatitis C virus infection requires the dynamic microtubule network: role of the viral nucleocapsid protein. J Biol Chem 284: 13778–13869. 19269968 10.1074/jbc.M807873200 PMC2679479

[64] Murray CL, Jones CT, Tassello J, Rice CM (2007) Alanine scanning of the hepatitis C virus core protein reveals numerous residues essential for production of infectious virus. J Virol 81: 10220–10231. 17634240 10.1128/JVI.00793-07 PMC2045476

[65] White SH, Wimley WC (1998) Hydrophobic interactions of peptides with membrane interfaces. Biochim Biophys Acta 1376: 339–352. 9804985 10.1016/s0304-4157(98)00021-5

[66] Counihan N, Rawlinson S, Lindenbach B (2011) Trafficking of hepatitis C virus core protein during virus particle assembly. PLoS Pathog 7: e1002302. 22028650 10.1371/journal.ppat.1002302 PMC3197604

[67] Shubeita GT, Tran SL, Xu J, Vershinin M, Cermelli S, et al (2008) Consequences of motor copy number on the intracellular transport of kinesin-1-driven lipid droplets. Cell 135: 1098–1107. 19070579 10.1016/j.cell.2008.10.021 PMC2768369

[68] Suomalainen M, Nakano MY, Boucke K, Keller S, Greber UF (2001) Adenovirus-activated PKA and p38/MAPK pathways boost microtubule-mediated nuclear targeting of virus. EMBO J 20: 1310–1319. 11250897 10.1093/emboj/20.6.1310 PMC145525

[69] Erhardt A, Hassan M, Heintges T, Häussinger D (2002) Hepatitis C Virus Core Protein Induces Cell Proliferation and Activates ERK, JNK, and p38 MAP Kinases Together with the MAP Kinase Phosphatase MKP-1 in a HepG2 Tet-Off Cell Line. Virology 292: 272–284. 11878930 10.1006/viro.2001.1227

[70] Hayashi J, Aoki H, Kajino K, Moriyama M, Arakawa Y, et al (2000) Hepatitis C virus core protein activates the MAPK/ERK cascade synergistically with tumor promoter TPA, but not with epidermal growth factor or transforming growth factor α. Hepatology 32: 958–961. 11050045 10.1053/jhep.2000.19343

[71] Spaziani A, Alisi A, Sanna D, Balsano C (2006) Role of p38 MAPK and RNA-dependent Protein Kinase (PKR) in Hepatitis C Virus Core-dependent Nuclear Delocalization of Cyclin B1. J Biol Chem 281: 10983–10989. 16446363 10.1074/jbc.M512536200

[72] Wileman T (2006) Aggresomes and Autophagy Generate Sites for Virus Replication. Science 312: 875–878. 16690857 10.1126/science.1126766

[73] Wileman T (2007) Aggresomes and Pericentriolar Sites of Virus Assembly: Cellular Defense or Viral Design? Annu Rev Microbiol 61: 149–167. 17896875 10.1146/annurev.micro.57.030502.090836

[74] Neveu G, Barouch-Bentov R, Ziv-Av A, Gerber D, Jacob Y, et al (2012) Identification and Targeting of an Interaction between a Tyrosine Motif within Hepatitis C Virus Core Protein and AP2M1 Essential for Viral Assembly. PLoS Pathog 8: e1002845. 22916011 10.1371/journal.ppat.1002845 PMC3420927

[75] Harris C, Herker E, Farese RV, Ott M (2011) Hepatitis C Virus Core Protein Decreases Lipid Droplet Turnover. J Biol Chem 286: 42615–42625. 21984835 10.1074/jbc.M111.285148 PMC3234948

[76] Lyn RK, Kennedy DC, Sagan SM, Blais DR, Rouleau Y, et al (2009) Direct imaging of the disruption of hepatitis C virus replication complexes by inhibitors of lipid metabolism. Virology 394: 130–172. 19747705 10.1016/j.virol.2009.08.022

[77] Mazumder N, Lyn RK, Singaravelu R, Ridsdale A, Moffatt DJ, et al (2013) Fluorescence Lifetime Imaging of Alterations to Cellular Metabolism by Domain 2 of the Hepatitis C Virus Core Protein. PLoS One 8: e66738. 23826122 10.1371/journal.pone.0066738 PMC3691201

