# Supplementary figures and images for: Bidirectional Lipid Droplet Velocities Are Controlled by Differential Binding Strengths of HCV Core DII Protein
Source: PLoS One. 2013 Nov 1;8(11):e78065. doi: 10.1371/journal.pone.0078065 (PMC3815211; doi:10.1371/journal.pone.0078065)

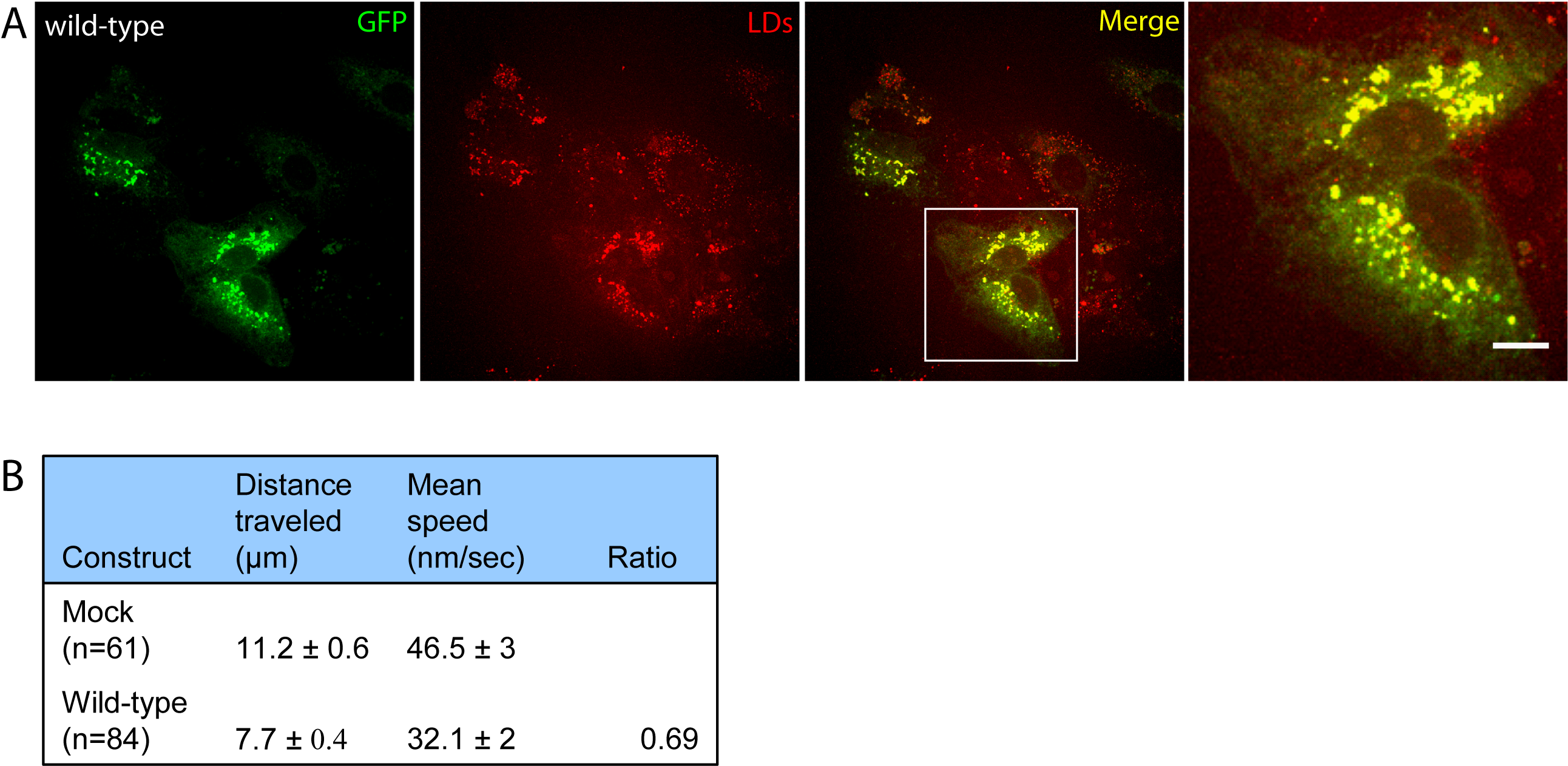

Supplement: Figure S1 — Particle tracking DII-corewt coated LDs in Huh-7 cells stably expressing an HCV subgenomic replicon. (A) CARS and TPF microscopy captures colocalization between DII-corewt and LDs, and captures DII-corewt-induced LD localization at the perinuclear region. Panel 4 is a magnified image selected by a region of interest from the merged image to project a clearer view of colocalization between DII-corewt and LDs. (B) Particle tracking DII-corewt coated LDs and LDs in mock cells not expressing DII-corewt. The overall mean travel distance and mean speeds were measured. The ratio is calculated by dividing the mean speed of DII-corewt coated LDs by LDs from the mock sample. The n represents the number of LDs that were particle tracked. Live-cell imaging was conducted for duration of four minutes with each frame interval acquired at 1.65 sec/frame. All scale bars represent 10 µm. (TIF) [file pone.0078065.s001.tif]

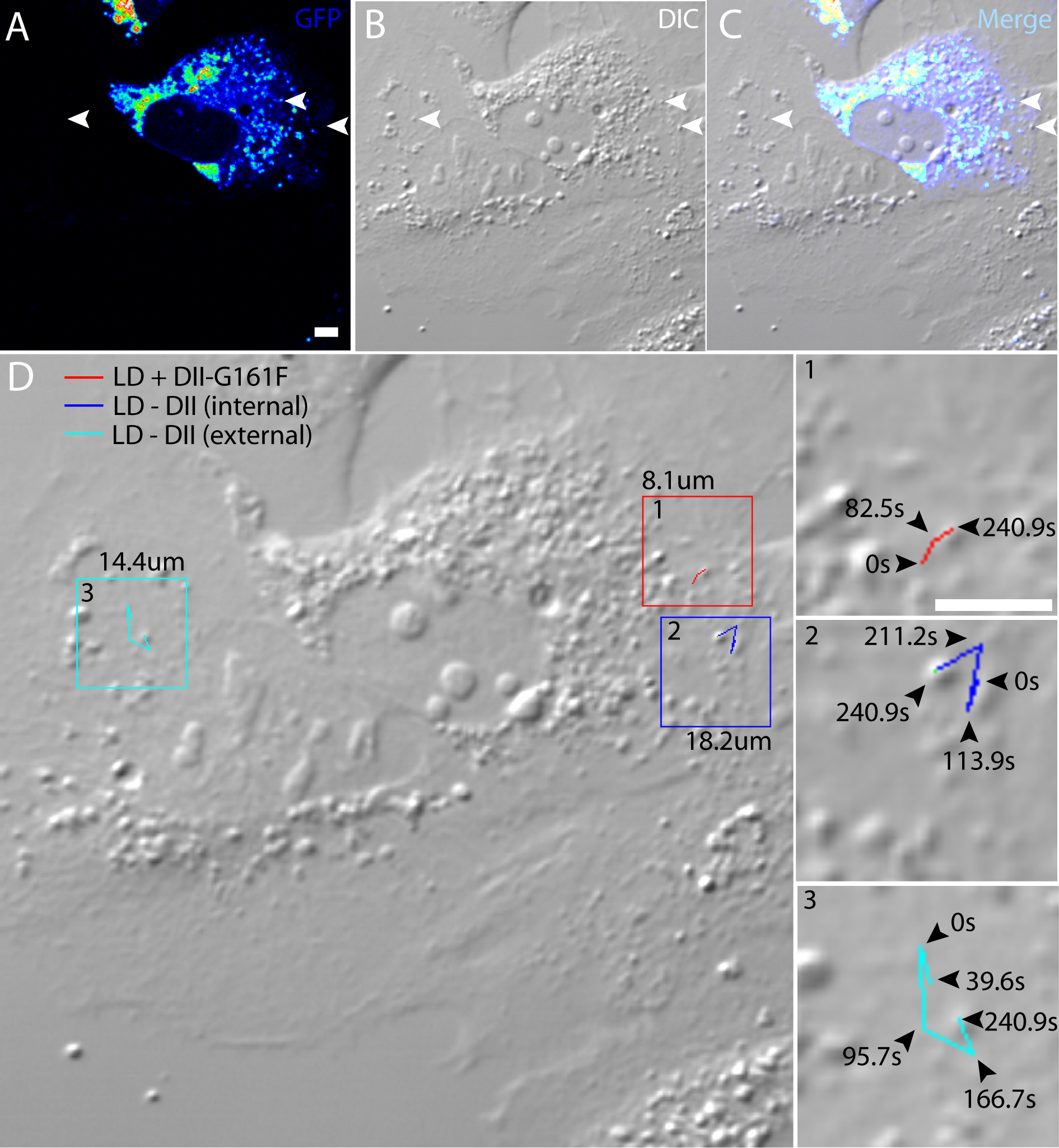

Supplement: Figure S2 — DII-coreG161F coated LDs are particle tracked using simultaneous TPF and DIC microscopy. This is a representative image of DII-coreG161F expressed in Huh-7 cells. Three individual LDs with dissimilar environments were selected (A–C, white arrows), and their trajectories were measured to calculate the overall distances traveled. (D) A larger DIC image of (B) includes boxes to identify each LD trajectory (inset 1–3). The value above each box (D) indicates their overall travel distances for (1) DII-coreG161F coated LD (2) non DII-coreG161F coated LD within the same cell, (3) and a LD in an adjacent cell not expressing DII-coreG161F. Each LD trajectory is magnified to demonstrate the LD track with selective freeze frame time-intervals representing the LD position at their indicated times. Due to frequent bidirectional movements, the displayed trajectories represent a general movement path, and does not portray total distance. All of the LDs are tracked according to the same start and end time. All scale bars represent 10 µm. (TIF) [file pone.0078065.s002.tif]

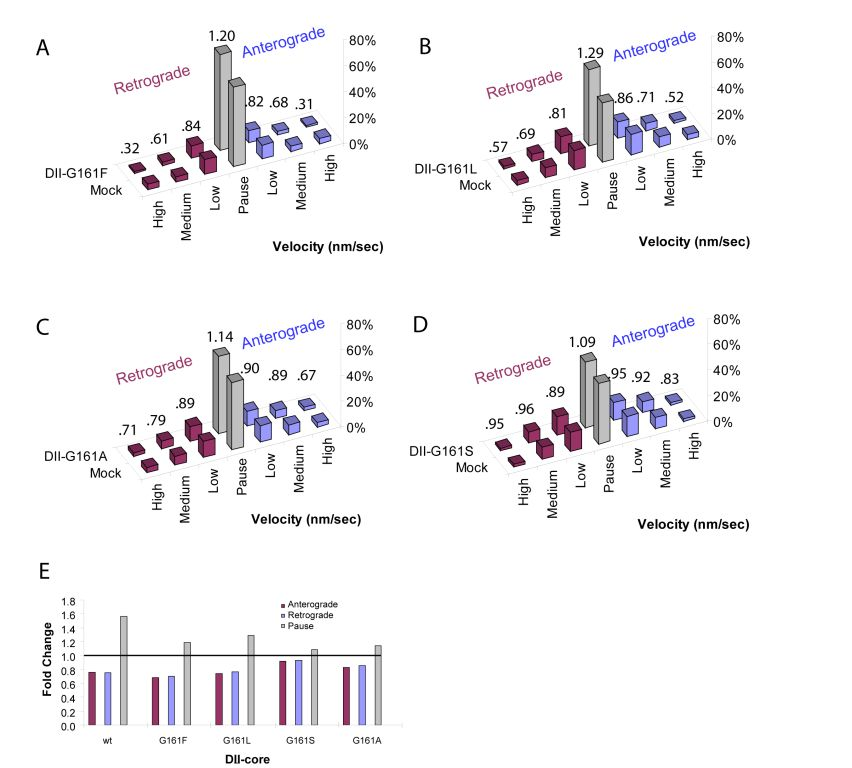

Supplement: Figure S3 — LD velocities are measured in Huh-7 cells expressing DII-core161 mutants. (A–D) The frequency of pauses (<15.7 nm/sec), low (15.7 nm/sec –50 nm/sec), medium (50.1 nm/sec –180 nm/sec), and high velocity (>180.1 nm/sec) measurements, expressed as a percentage, in both directions are plotted for LDs in cells expressing (A) DII-coreG161F, (B) DII-coreG161L, (C) DII-coreG161A, (D) DII-coreG161S. The ratios above each set of columns is calculated by dividing the frequency for each velocity interval of DII-core coated LDs by their respective mock LDs. (E) The total frequency of retrograde, anterograde, and pauses were also collected and presented as a fold-change measurement that compared LDs in all DII-core161 mutants with each of their respective mocks. (TIF) [file pone.0078065.s003.tif]

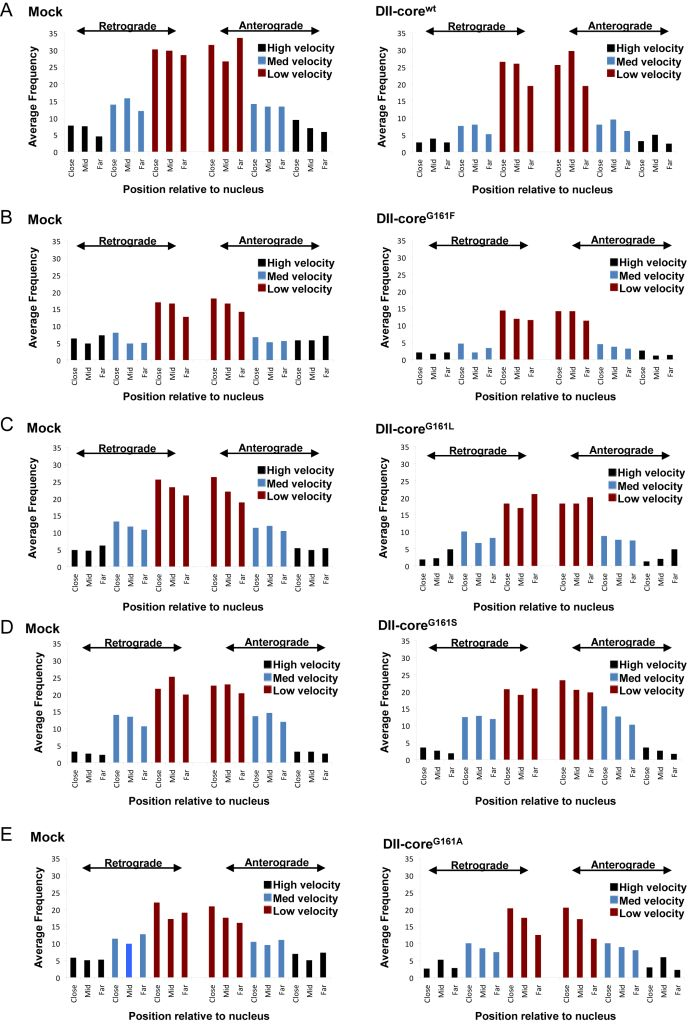

Supplement: Figure S4 — Frequency of LD velocities at three different regions in Huh-7 cells expressing DII-coremut and in the mock. The average frequency of low, medium, and high velocity runs for each direction was calculated for LDs bound to (A) DII-corewt, (B) DII-coreG161F, (C) DII-coreG161L, (D) DII-coreG161S, (E) DII-coreG161A. The data was separated according to where the LD was located at a position that was relative to the nucleus, either at a close, medium, or far location. (TIF) [file pone.0078065.s004.tif]

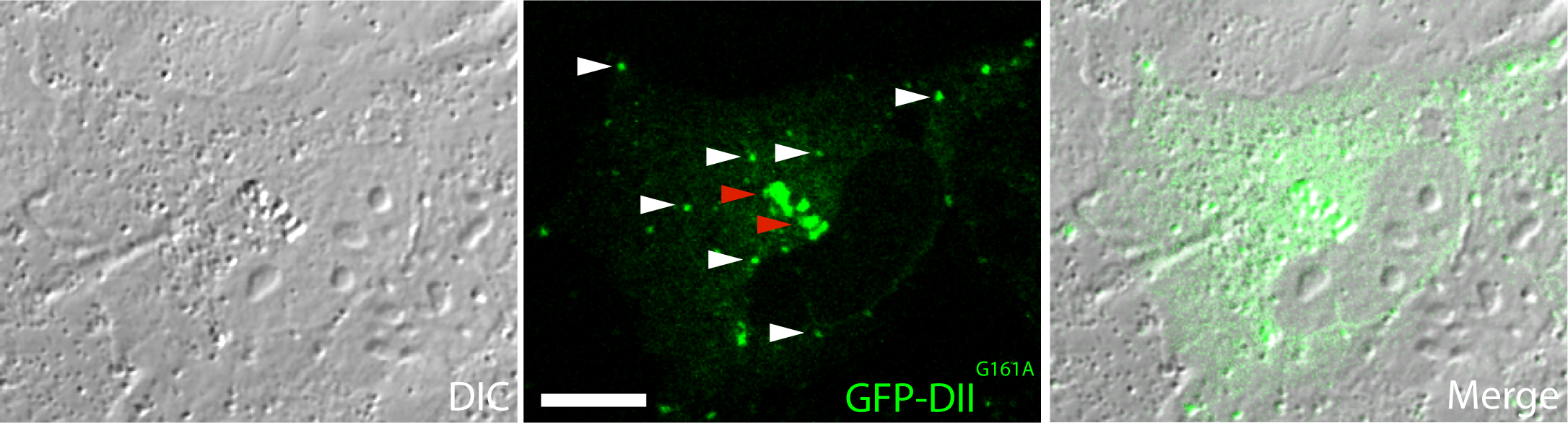

Supplement: Figure S5 — Two populations of DII-coreG161A coated LDs was observed. This is a representative image with a pattern that is typically observed in all other DII-core constructs. The white arrow represents a LD population of individual LDs that are bound to DII-coreG161A. The red arrow corresponds to tightly packed LDs with a high abundance of DII-coreG161A colocalized at the same region. Individual LDs are indistinguishable at this region. All scale bars represent 10 µm. (TIF) [file pone.0078065.s005.tif]
